# *STIM1*/*ORAI1* Loss-of-Function and Gain-of-Function Mutations Inversely Impact on SOCE and Calcium Homeostasis and Cause Multi-Systemic Mirror Diseases

**DOI:** 10.3389/fphys.2020.604941

**Published:** 2020-11-04

**Authors:** Roberto Silva-Rojas, Jocelyn Laporte, Johann Böhm

**Affiliations:** Institut de Génétique et de Biologie Moléculaire et Cellulaire (IGBMC), Inserm U1258, CNRS UMR 7104, Université de Strasbourg, Illkirch, France

**Keywords:** SOCE, calcium, STIM1, ORAI1, CRAC channelopathy, tubular aggregate myopathy, Stormorken syndrome

## Abstract

Store-operated Ca^2+^ entry (SOCE) is a ubiquitous and essential mechanism regulating Ca^2+^ homeostasis in all tissues, and controls a wide range of cellular functions including keratinocyte differentiation, osteoblastogenesis and osteoclastogenesis, T cell proliferation, platelet activation, and muscle contraction. The main SOCE actors are STIM1 and ORAI1. Depletion of the reticular Ca^2+^ stores induces oligomerization of the luminal Ca^2+^ sensor STIM1, and the oligomers activate the plasma membrane Ca^2+^ channel ORAI1 to trigger extracellular Ca^2+^ entry. Mutations in *STIM1* and *ORAI1* result in abnormal SOCE and lead to multi-systemic disorders. Recessive loss-of-function mutations are associated with CRAC (Ca^2+^ release-activated Ca^2+^) channelopathy, involving immunodeficiency and autoimmunity, muscular hypotonia, ectodermal dysplasia, and mydriasis. In contrast, dominant *STIM1* and *ORAI1* gain-of-function mutations give rise to tubular aggregate myopathy and Stormorken syndrome (TAM/STRMK), forming a clinical spectrum encompassing muscle weakness, thrombocytopenia, ichthyosis, hyposplenism, short stature, and miosis. Functional studies on patient-derived cells revealed that CRAC channelopathy mutations impair SOCE and extracellular Ca^2+^ influx, while TAM/STRMK mutations induce excessive Ca^2+^ entry through SOCE over-activation. In accordance with the opposite pathomechanisms underlying both disorders, CRAC channelopathy and TAM/STRMK patients show mirror phenotypes at the clinical and molecular levels, and the respective animal models recapitulate the skin, bones, immune system, platelet, and muscle anomalies. Here we review and compare the clinical presentations of CRAC channelopathy and TAM/STRMK patients and the histological and molecular findings obtained on human samples and murine models to highlight the mirror phenotypes in different tissues, and to point out potentially undiagnosed anomalies in patients, which may be relevant for disease management and prospective therapeutic approaches.

## Introduction

Calcium (Ca^2+^) is an elemental factor regulating a multitude of metabolic processes, signaling pathways, and cellular functions in all tissues, and mediates muscle contraction, nerve conduction, hormone release, and blood coagulation. Consistently, normal tissue and organ physiology strictly depends on the precise control of Ca^2+^ entry, storage, and release, while abnormal Ca^2+^ homeostasis induces various rare and common disorders affecting skeletal muscle, heart, bones, brain, skin, or the immune and hormonal systems (Peacock, 2010; Gattineni, 2014).

Ca^2+^ is mainly stored in the endoplasmic/sarcoplasmic reticulum (ER/SR), and refilling of the stocks is initiated by store-operated Ca^2+^ entry (SOCE), a ubiquitous mechanism driven by the concerted action of STIM1 and ORAI1 (Zhang et al., 2005; Feske et al., 2006). STIM1 contains an intraluminal region with EF hands sensing the reticular Ca^2+^ concentration, and a cytosolic part interacting with the plasma membrane CRAC (Ca^2+^ release-activated Ca^2+^) channel ORAI1 (Stathopulos et al., 2006, 2008). Ca^2+^ store depletion induces STIM1 unfolding and oligomerization, and the STIM1 oligomers hence activate ORAI1 to trigger extracellular Ca^2+^ entry (Stathopulos et al., 2008; Prakriya and Lewis, 2015; Stathopulos and Ikura, 2017).

**FIGURE 1:**
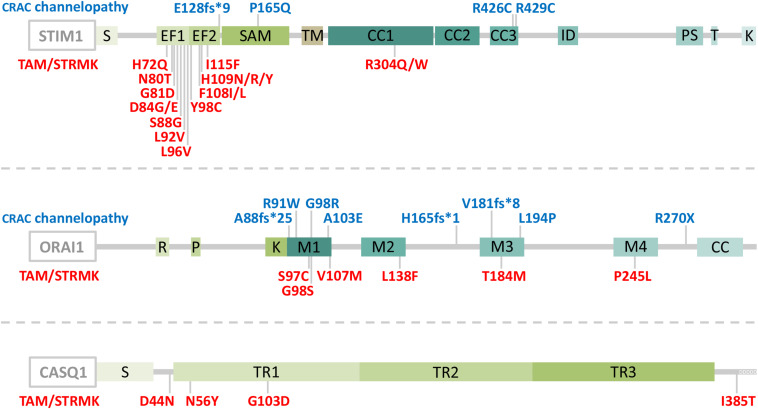
Schematic representation of STIM1, ORAI1, and CASQ1 with position of the CRAC channelopathy and TAM/STRMK mutations. STIM1 is composed of a luminal part with a reticular signal sequence (S), Ca^2+^-binding EF-hands and a SAM domain, a transmembrane domain (TM), and a cytosolic part with coiled-coil (CC) domains 1–3, an inhibitory domain (ID), a proline/serine-rich region (PS), a TRIP domain (T), and a lysine-rich region (K). ORAI1 contains arginine (R), proline (P), and lysine (K)-rich regions, four transmembrane domains (M1-M4), and a coiled-coil domain (CC), and calsequestrin (CASQ1) contains a reticular signal sequence (S), three thioredoxin domains (TR1-3), and an N-terminal stretch of Ca^2+^-binding aspartic acid residues (DDDDD). CRAC channelopathy mutations are depicted in blue and TAM/STRMK mutations in red. Note that an additional *STIM1* splice site mutation (c.970-1G>A) generates unstable transcripts and causes CRAC channelopathy.

Abnormal SOCE has been associated with different human disorders. Recessive *STIM1* and *ORAI1* loss-of-function (LoF) mutations resulting in insufficient SOCE cause CRAC channelopathies characterized by severe combined immunodeficiency (SCID) involving recurrent and chronic infections, autoimmunity, muscular hypotonia, ectodermal dysplasia, anhidrosis, and mydriasis (Feske et al., 2006; Picard et al., 2009; Lacruz and Feske, 2015). The majority of the LoF mutations involve a total loss of STIM1 or ORAI1 (Lacruz and Feske, 2015), but single point mutations disrupting the STIM1 function and interfering with the STIM1-ORAI1 interaction (R426C, R429C) (Fuchs et al., 2012; Wang et al., 2014) or generating an obstructed ORAI1 channel (R91W) (Feske et al., 2006) have also been described (Figure 1). In contrast, dominant *STIM1* and *ORAI1* gain-of-function (GoF) mutations inducing excessive Ca^2+^ entry through SOCE over-activation were found in patients with tubular aggregate myopathy (TAM) and Stormorken syndrome (STRMK) (Bohm et al., 2013; Misceo et al., 2014; Morin et al., 2014; Nesin et al., 2014). TAM and STRMK form a clinical continuum characterized by progressive muscle weakness and myalgia predominantly affecting the lower limbs (Chevessier et al., 2005), and most patients manifest a varying degree of additional multi-systemic signs as miosis, ichthyosis, short stature, hyposplenism, thrombocytopenia, and dyslexia (Endo et al., 2015; Markello et al., 2015; Walter et al., 2015; Bohm et al., 2017; Garibaldi et al., 2017; Noury et al., 2017; Bohm and Laporte, 2018; Morin et al., 2020). All GoF mutations are missense mutations affecting highly conserved amino acids in the Ca^2+^-binding EF hands (H72Q, N80T, G81D, D84G, D84E, S88G, L92V, L96V, Y98C, F108I, F108L; H109N, H109R, H109Y, I115F) (Bohm et al., 2013, 2014; Hedberg et al., 2014; Markello et al., 2015; Walter et al., 2015; Harris et al., 2017; Noury et al., 2017; Li et al., 2019; Claeys et al., 2020; Morin et al., 2020) or in the luminal coiled-coil domains of STIM1 (R304W, R304Q) (Misceo et al., 2014; Morin et al., 2014; Nesin et al., 2014; Markello et al., 2015; Harris et al., 2017; Alonso-Jimenez et al., 2018; Borsani et al., 2018; Sura et al., 2020), or in the ORAI1 transmembrane domains forming the channel pore or concentric rings surrounding the pore (G97C, G98S, V107M, L138F, T184M, P245L) (Nesin et al., 2014; Endo et al., 2015; Bohm et al., 2017; Garibaldi et al., 2017; Figure 1). Missense mutations in the muscle-specific SR Ca^2+^ buffer calsequestrin (*CASQ1*) have moreover been reported in patients with late-onset muscle weakness and myalgia, forming the mild end of the TAM/STRMK spectrum (Barone et al., 2017; Bohm et al., 2018; Figure 1).

Animal models for CRAC channelopathy and TAM/STRMK exist and widely recapitulate the clinical signs of the human disorders. Mice lacking STIM1 or ORAI1 die perinatally (Baba et al., 2008; Oh-Hora et al., 2008), and the tissue-specific deletion of *Stim1* or *Orai1* or the generation of chimeras through transplantation of hematopoietic *Stim1^–/–^* or *Orai1^–/–^* stem cells results in defective T cell activation and Treg suppression (Gwack et al., 2008; Oh-Hora et al., 2008, 2013; McCarl et al., 2010), splenomegaly (Oh-Hora et al., 2008, 2013), autoimmunity (Oh-Hora et al., 2008, 2013), reduced platelet activation and thrombus formation (Varga-Szabo et al., 2008; Bergmeier et al., 2009; Braun et al., 2009; Ahmad et al., 2011), anhidrosis (Concepcion et al., 2016), amelogenesis imperfecta (Gwack et al., 2008), and muscle weakness with reduced resistance to fatigue (Stiber et al., 2008; Srikanth et al., 2010; Li et al., 2012; Wei-Lapierre et al., 2013; Carrell et al., 2016; Sampieri et al., 2018). Mice harboring the *Stim1* GoF mutations D84G, I115F, or R304W show a varying degree of multi-systemic disease signs including small size (Cordero-Sanchez et al., 2019; Silva-Rojas et al., 2019), eye movement defects (Silva-Rojas et al., 2019), skin and spleen anomalies (Grosse et al., 2007; Cordero-Sanchez et al., 2019; Silva-Rojas et al., 2019), bleeding diathesis with thrombocytopenia (Grosse et al., 2007; Cordero-Sanchez et al., 2019; Silva-Rojas et al., 2019), and muscle weakness (Cordero-Sanchez et al., 2019; Silva-Rojas et al., 2019). SOCE deficiency in drosophila resulting from *Stim* or *Orai* downregulation impairs the flight capacities (Venkiteswaran and Hasan, 2009; Agrawal et al., 2010), and zebrafish embryos injected with mRNA containing *STIM1* or *ORAI1* GoF mutations display thrombocytopenia (Nesin et al., 2014), highlighting the conservation of SOCE in specific tissues.

**FIGURE 2:**
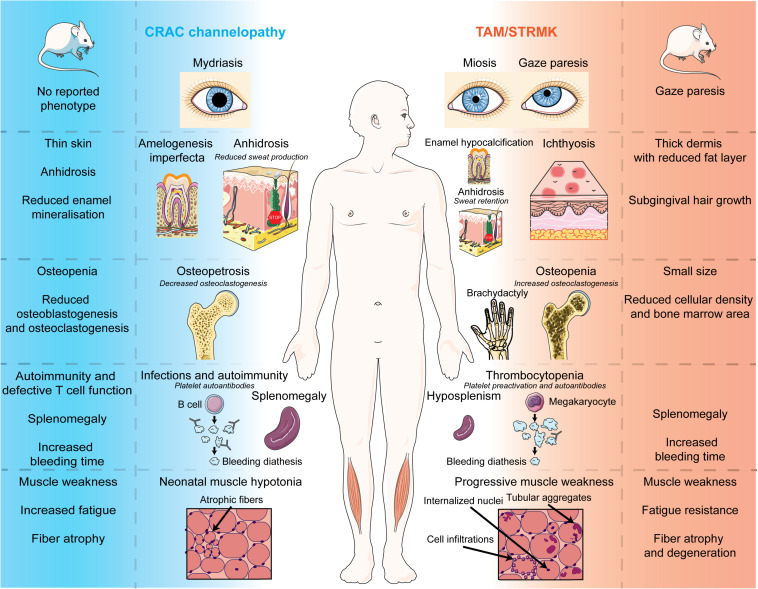
Phenotypes of CRAC channelopathy and TAM/STRMK in patients and mouse models. Schematic overview of the clinical and molecular phenotypes of eyes, skin, teeth, spleen, immune system, and skeletal muscle in CRAC channelopathy and TAM/STRMK. The figure uses modified images from Servier Medical Art Commons Attribution 3.0 Unported License (http://smart.servier.com).

The present review aims to provide an update on the current knowledge of CRAC channelopathy and TAM/STRMK, to highlight the molecular and/or clinical mirror phenotypes caused by either LoF or GoF mutations in the SOCE key players, and to provide an overview of the available animal models recapitulating the human disorders. We thoroughly and stepwise compare the eye, skin, bone, enamel, spleen, immune, platelet, and muscle phenotypes in human and mouse, and we detail the inverse mutational effects and pathomechanisms underlying CRAC channelopathy and TAM/STRMK, and their impact on the sequence of events leading to the diverging clinical manifestations and mirror-image anomalies in most affected tissues. We also point to clinical signs that are potentially underdiagnosed in patients, and may be relevant for diagnosis and disease management, and disclose treatment options. A schematic illustration opposing the clinical pictures of CRAC channelopathy versus TAM/STRMK is shown in Figure 2, and is supported by a detailed description in Table 1.

**TABLE 1:** Descriptive comparison of the clinical signs and physiological defects in CRAC channelopathy and TAM/STRMK patients and mouse models.

		CRAC channelopathy Reduced SOCE		TAM/STRMK Increased SOCE	
		
		Mouse models	Patients	Patients	Mouse models
Eye	Pupils	Not reported	Mydriasis	Miosis	Not reported
	Eye movement	Not reported	Not reported	Upward/lateral gaze paresis	Upward gaze paresis (*Stim1^*R*304W/+^*)
Ectodermal tissues	Skin	Thin skin (*Orai1^–/–^*), anhidrosis (*Orai1^–/–^*, *Stim1^–/–^Stim2^–/–^*)	Anhidrosis	Anhidrosis, ichthyosis	Thick dermis, reduced subcutaneous fat layer (*Stim1^*R*304W/+^*)
	Teeth	Reduced enamel mineralization (*Orai1^–/–^*)	Amelogenesis imperfecta	Enamel hypocalcification	Subgingival hair growth (*Stim1^*R*304W/+^*)
Bones	Clinical signs	Not reported	Facial dysmorphism	Short stature brachydactyly, syndactyly, Klippel-Feil anomaly	Small size (*Stim1^*R*304W/+^*), reduced number of ribs (*Stim1^*R*304W/R304W^*), thin and compact bones (*Stim1^*R*304W/R304W^*)
	Molecular findings	Reduced osteoblastogenesis and osteoclastogenesis, osteopenia (*Orai1^–/–^*)	Osteopetrosis, reduced osteoclasteogenesis	Osteopenia, increased osteoclasteogenesis	Reduced cellular density, reduced bone marrow area (*Stim1^*R*304W/+^*)
Immune system	Clinical signs	Autoimmunity, splenomegaly (*Stim1^–/–^Stim2^–/–^, Orai1^*R*93W/R93W^*)	Recurrent and chronic infections, autoimmunity, splenomegaly	Hyposplenism	Splenomegaly (*Stim1^*D*84G/+^*, *Stim1^*I*115F/+^* and *Stim1^*R*304W/+^*)
	Molecular findings	Reduced cytokine expression (*Stim1^–/–^*, *Orai1i^–/–^*, *Stim1^–/–^Stim2^–/–^, Orai1^*R*93W/R93W^*), reduced suppressive function of NKT and Treg cells (*Stim1^–/–^Stim2^–/–^, Orai1^*R*93W/R93W^*)	Reduced T cell proliferation, cytokine expression, immunoglobulin production, iNKT and Treg cells, presence of anti-platelet autoantibodies	Lymphoproliferation, presence of anti-platelet autoantibodies	Reduced Treg and NK cells, increased neutrophils and monocytes (*Stim1^*R*304W/+^*)
Coagulation	Clinical signs	Slightly increased bleeding time (*Stim1^–/–^*, *Orai1^–/–^*)	Mild bleeding diathesis	Bleeding diathesis	Increased bleeding time (*Stim1^*D*84G/+^*)
	Molecular findings	Reduced platelet activation (*Stim1^–/–^*, *Orai1^–/–^*, *Stim1^–/–^Orai1^–/–^*, *Orai1^*R*93W/R93W^*), reduced thrombus formation (*Stim1^–/––^*, *Orai1^–/–^*, *Stim1^–/–^Orai1^–/–^*)	Reduced platelet activation, reduced thrombus formation	Thrombocytopenia, Platelet pre-activation, aberrant size and morphology, reduced platelet-platelet adhesion	Thrombocytopenia (*Stim1^*D*84G/+^, Stim1^*I*115F/+^, Stim1^*R*304W/+^*), platelet pre-activation, increased platelet clearance (*Stim1^*D*84G/+^*)
Skeletal muscle	Clinical signs	Muscle weakness (*Stim1^–/–^*, *Orai1^–/–^*), increased fatigue (*Stim1^–/–^*, *Orai1^–/–^*)	Neonatal hypotonia, muscle weakness	Muscle weakness, cramps, myalgia	Muscle weakness, increased resistance to fatigue (*Stim1^*I*115F/+^*, *Stim1^*R*304W/+^*)
	Molecular findings	Fiber atrophy, swollen mitochondria (*Stim1^–/–^*, *Orai1^–/–^*)	Type I predominance, type II fiber atrophy	Tubular aggregates, type I fiber predominance, type II fiber atrophy, internalized nuclei, vacuoles, fibrosis, elevated serum CK	Fiber atrophy and degeneration, type I fiber predominance, swollen mitochondria, elevated serum CK (*Stim1^*I*115F/+^*, *Stim1^*R*304W/+^*)

## Phenotypic Traits in CRAC Channelopathy and TAM/STRMK Patients and Mice

CRAC channelopathy and TAM/STRMK are multi-systemic disorders, and patients with either disease can manifest impairments of pupillary function, eye movement, skin, enamel, bones, immune system, spleen, coagulation, and skeletal muscle. The following chapter provides a comparative overview of the clinical anomalies and the molecular and mechanistic causes, and also refers to the phenotypic traits of diverse CRAC channelopathy and TAM/STRMK mouse models.

### Pupillary Dysfunction and Eye Movement Limitations

Vision is primarily a photochemical process, and can be adapted to the lighting conditions through iris constriction/dilatation and eye movement, both governed by Ca^2+^-dependent muscle contraction. Ca^2+^ release from the reticulum activates the contractile apparatus, which generates force, causing the shortening of the muscle cells (Ebashi, 1974). The iris acts as a diaphragm controlling the amount of light entering the eye through the pupil, and SOCE substantially sustains the muscle tonus for the steady contraction of the smooth sphincter and dilator muscles for an appropriate view in brightness and obscurity (Eckstein et al., 2017; Feldman et al., 2017).

Pupillary dysfunction is a main clinical sign of CRAC channelopathy and TAM/STRMK. While CRAC channelopathy patients typically show iris dilatation (mydriasis) (Feske et al., 2006; Picard et al., 2009; Fuchs et al., 2012; Lian et al., 2018), the inverse phenotype of light-insensitive iris hypercontraction (miosis) is a hallmark of TAM/STRMK, and results in migraine and reduced night vision (Misceo et al., 2014; Morin et al., 2014, 2020; Nesin et al., 2014; Markello et al., 2015; Bohm et al., 2017; Garibaldi et al., 2017; Harris et al., 2017; Alonso-Jimenez et al., 2018; Borsani et al., 2018; Claeys et al., 2020; Sura et al., 2020). Mydriasis and miosis have, however, not been described in murine models for CRAC channelopathy or TAM/STRMK. They may have been missed, or may reflect physiological differences between the species. Indeed, mice are nocturnal animals, and murine pupil constriction is essentially triggered by a light-dependent mechanism known as local pupillary reflex that is absent in humans (Xue et al., 2011).

Eye movement relies on the rapid and concerted contraction of six striated extraocular muscles, and ophthalmoplegia including upward gaze paresis (Bohm et al., 2013; Noury et al., 2017), lateral gaze paresis (Morin et al., 2020), or reduced lateral and/or upward gaze (Bohm et al., 2014; Hedberg et al., 2014; Markello et al., 2015; Walter et al., 2015; Harris et al., 2017; Noury et al., 2017) is commonly seen in TAM/STRMK patients. In accordance, the TAM/STRMK mouse model harboring the most common STIM1 GoF mutation R304W also features an upward gaze paresis (Silva-Rojas et al., 2019).

### Skin Anomalies and Enamel Defects

Skin forms the first defense barrier to protect from external agents, and also plays a pivotal role in thermoregulation by sweat production. Keratinocytes are the principal components of the outermost skin layer, the epidermis, and their growth, differentiation, and migration is driven by SOCE in both humans and mice (Numaga-Tomita and Putney, 2013; Vandenberghe et al., 2013). SOCE also triggers the opening of the Ca^2+^-activated chloride channel TMEM16A in the sweat glands, and thereby enables chloride secretion and sweat production (Concepcion et al., 2016). In the absence of SOCE, CRAC channelopathy patients present with thermoregulatory instability and anhidrosis accompanied by heat intolerance, dry skin, and eczema (Feske et al., 2006; Fuchs et al., 2012; Schaballie et al., 2015; Lian et al., 2018). Skin anomalies including eczema and ichthyosis are also commonly seen in TAM/STRMK patients (Misceo et al., 2014; Morin et al., 2014, 2020; Bohm et al., 2017; Harris et al., 2017; Claeys et al., 2020), and one patient additionally manifested anhidrosis (Ishitsuka et al., 2019). Histological examinations of the skin biopsy revealed an obstruction of the spiral duct in the eccrine gland, the acrosyringia, resulting in sweat retention. This is different from CRAC channelopathy patients, where the sweat glands display a reduced lumen due the lack of sweat production (Lian et al., 2018). Noteworthy, the ectodermal barrier protein proflaggrin was found to be aggregated in the acrosyringia of the TAM/STRMK patient. Loss of proflaggrin is a major predisposing factor of idiopathic ichthyosis (Palmer et al., 2006), indicating that the skin phenotype in TAM/STRMK patients may be a direct consequence of the abnormal proflaggrin accumulation in the sweat glands.

Alike skin and sweat glands, teeth derive from the ectoderm, and CRAC channelopathy patients also manifest dental maturation defects including major enamel loss, discoloration and poor mineralization of both deciduous and permanent teeth (Feske et al., 2006; McCarl et al., 2009; Picard et al., 2009; Fuchs et al., 2012; Wang et al., 2014; Schaballie et al., 2015; Lian et al., 2018), highlighting the importance of SOCE in ameloblast formation and mineralization (Wang et al., 2014). In contrast, amelogenesis imperfecta is not a typical feature of TAM/STRMK, and enamel hypocalcification was only noted in a single patient (Noury et al., 2017).

CRAC channelopathy and TAM/STRMK animal models partially recapitulate the human enamel and skin phenotypes. Mice lacking ORAI1 manifest reduced enamel mineralization (Robinson et al., 2012) and thinner skin with elongated keratinocytes and smaller vibrissae follicles (Gwack et al., 2008), and the ectodermal-specific knockout of *Orai1* or *Stim1*/*Stim2* impairs SOCE and results in anhidrosis and a reduced sweat gland lumen (Concepcion et al., 2016). The TAM/STRMK mouse harboring the STIM1 R304W mutation shows a thickened dermis and a reduction of the subcutaneous fat layer (Silva-Rojas et al., 2019), and a subset of the animals additionally exhibit subgingival hair growth on the lower incisors (Gamage et al., 2020).

### Bone Anomalies

Bones represent 15% of the total body weight and are essential for motion, mineral storage, and hematopoiesis. Bone deposition and resorption are dynamic and balanced processes driven by bone-forming osteoblasts and bone-resorbing osteoclasts (Florencio-Silva et al., 2015), and their growth and differentiation is regulated by SOCE-dependent Ca^2+^ homeostasis (Eapen et al., 2010; Blair et al., 2011; Chen et al., 2018). Bone resorption by osteoclasts generates a local increment of extracellular Ca^2+^, inducing the activation of the calcineurin/NFAT signaling pathway, and resulting in osteoblastogenesis (Zayzafoon, 2006). Calcineurin/NFAT signaling is also essential for osteoclastogenesis and T cell activation, and the inhibition of this pathway with cyclosporine A to prevent transplant rejection is associated with an increased incidence of bone fractures (Zayzafoon, 2006).

Overt bone anomalies are largely absent in CRAC channelopathy and TAM/STRMK patients with exception of individual cases with facial dysmorphism (McCarl et al., 2009), fusion of the cervical vertebrae (Klippel-Feil anomaly) (Morin et al., 2020), brachydactyly (Morin et al., 2020), or syndactyly of the second and third toes (Borsani et al., 2018). Bone mineralization was found to be increased in two CRAC channelopathy patients (osteopetrosis) and decreased in a single TAM/STRMK patient (osteopenia), and accordingly, functional studies demonstrated a decreased osteoclastogenesis in bone marrow mononuclear macrophages from the CRAC channelopathy patients, and an increased osteoclastogenesis in cells derived from the TAM/STRMK patient (Huang et al., 2020). Of note, a number of TAM/STRMK patients exhibit a short stature (Misceo et al., 2014; Morin et al., 2014, 2020; Noury et al., 2017; Borsani et al., 2018), and other more subtle or late-onset bone disorders might have been overlooked in CRAC channelopathy and TAM/STRMK patients. This is supported by the impaired differentiation and function of osteoblasts and osteoclasts leading to osteopenia with decreased bone density and trabecular bone volume in ORAI1-deficient mice (Hwang et al., 2012; Robinson et al., 2012). Alike patients, TAM/STRMK mice are smaller than their littermates, and micro-CT analyses revealed a decreased cellular density and a reduced bone marrow area in femur and tibia, potentially affecting bone strength and stiffness (Silva-Rojas et al., 2019). Surviving mice carrying the STIM1 R304W mutation at the homozygous state show a more severe skeletal phenotype with thinner and more compact bones, and also feature a reduced number of ribs (Gamage et al., 2020).

### Immune System and Spleen Anomalies

The immune system is an essential and complex defense network, and SOCE directs the fate and function of diverse cells of the innate and adaptive immune system, including dendritic cell maturation (Felix et al., 2013), neutrophil activation (Zhang et al., 2014), lymphocyte cytotoxicity and cytokine production (Maul-Pavicic et al., 2011), as well as T cell proliferation, differentiation, and metabolism (Vaeth et al., 2017; Vaeth and Feske, 2018). T cells play a pivotal role in the adaptive immune system and act as effector, memory, suppressor, or helper cells in response to external agents. The antigen recognition by the T cell receptors activates a signaling cascade resulting in the continuous depletion of the reticular Ca^2+^ stores and a durable extracellular Ca^2+^ entry via SOCE to initiate the Ca^2+^-dependent transcriptional program necessary for T cell function (Feske, 2007).

Recurrent infections and autoimmunity are the predominant clinical traits of CRAC channelopathy (Feske et al., 2006; McCarl et al., 2009; Picard et al., 2009; Byun et al., 2010; Fuchs et al., 2012; Wang et al., 2014; Chou et al., 2015; Lacruz and Feske, 2015; Schaballie et al., 2015; Badran et al., 2016; Lian et al., 2018), and hematological examinations of affected individuals revealed normal levels of T cells, while functional investigations detected a reduced T cell proliferation and cytokine expression, and an impaired production of immunoglobulins in response to antigens (Feske et al., 1996, 2006; Fuchs et al., 2012; Lian et al., 2018). Invariant natural killer T cells (iNKT) and/or regulatory T cells (Treg) were reduced (Schaballie et al., 2015; Badran et al., 2016; Lian et al., 2018), suggesting a defect in self-tolerance as in autoimmune disorders (Dejaco et al., 2006; Novak and Lehuen, 2011; Josefowicz et al., 2012). In accordance with the immune cell dysregulation in patients, cytokine expression is impaired in mice with T CD4+ cell-specific deletion of *Stim1* or *Orai1*, and chimeric *Orai1^*R*93W/R93W^* animals (corresponding to R91W in humans) and *Stim1* and *Stim2* double knockout mice additionally show a reduced suppressive function of Treg and NKT cells and an associated autoimmunity and splenomegaly (Gwack et al., 2008; Oh-Hora et al., 2008, 2013; McCarl et al., 2010).

The spleen is the largest lymphoid organ and functions as a blood filter, and ensures the biogenesis and storage of white and red blood cells, as well as the phagocytosis of circulating microorganisms (de Porto et al., 2010). CRAC channelopathy patients develop hepatosplenomegaly (Picard et al., 2009; Byun et al., 2010; Schaballie et al., 2015; Lian et al., 2018), while asplenia or hyposplenia is a clinical hallmark of TAM/STRMK (Morin et al., 2020). As an indication of abnormal spleen function, Howell-Jolly bodies have moreover been found on peripheral blood film in several affected individuals (Misceo et al., 2014; Morin et al., 2014; Markello et al., 2015; Harris et al., 2017; Noury et al., 2017), but an increased rate of infections has nevertheless not been reported. Contrasting the patients, the TAM/STRMK mouse models present with splenomegaly, and histological investigations of the spleen revealed megakaryocyte hypoplasia (Grosse et al., 2007; Silva-Rojas et al., 2019). This is possibly related to a physiological difference between both species, as hematopoiesis lowers with age in humans, while it is maintained throughout life in mice (Bronte and Pittet, 2013). Of note, hematological analyses disclosed abnormal B, NK, and Treg counts in the STIM1 R304W mouse (Silva-Rojas et al., 2019), indicating that disturbances of the immune system may also occur in TAM/STRMK patients and potentially contribute to the spleen, platelet, and skin anomalies. This is sustained by the detection of lymphoproliferation and circulating antibodies against platelets in a single patient with STIM1 R304W mutation (Sura et al., 2020).

### Coagulation Defects

Hemostasis prevents and stops bleeding through the formation of a thrombus, which is ultimately resolved in the process of wound healing. Platelets play an essential role in thrombus formation, and the activation of platelets is induced by the presence of the subcortical component collagen in the blood flow following vessel wall damage (Bye et al., 2016). The collagen fragments bind to glycoprotein VI (GPVI) at the surface of the platelets and trigger a signaling cascade involving SOCE and leading to the Ca^2+^-dependent exposure of phosphatidylserine (PS) and the secretion of alpha granules containing thrombotic factors (Berna-Erro et al., 2016; van der Meijden and Heemskerk, 2019), which will then prompt the coagulation process and modulate inflammation and angiogenesis in the injured area (Blair and Flaumenhaft, 2009).

As a result of SOCE deficiency, PS exposure and alpha granule secretion is reduced in platelets from CRAC channelopathy patients, impeding platelet aggregation and thrombus formation (Nakamura et al., 2013). In consequence of the overall reduction of Treg cells, high titers of anti-platelet autoantibodies are detectable in the serum of the patients, lead to hemolytic anemia, and contribute to mild or intermittent susceptibility to bleed in several affected individuals (Picard et al., 2009; Byun et al., 2010; Fuchs et al., 2012; Lian et al., 2018). Bleeding diathesis associated with thrombocytopenia is a major clinical feature of TAM/STRMK (Misceo et al., 2014; Morin et al., 2014, 2020; Nesin et al., 2014; Markello et al., 2015; Bohm et al., 2017; Harris et al., 2017; Noury et al., 2017; Alonso-Jimenez et al., 2018; Borsani et al., 2018; Li et al., 2019; Claeys et al., 2020; Sura et al., 2020), and the analysis of blood samples from patients revealed increased platelet activation markers and enhanced secretion of alpha granules in unstimulated platelets (Misceo et al., 2014). Despite this pre-activation state caused by elevated resting Ca^2+^, the platelet-platelet adhesion is impaired, and platelets often appeared with aberrant size and morphology (Markello et al., 2015), suggesting that the coagulation defect in TAM/STRMK patients results from a combination of platelet loss and platelet dysfunction.

In analogy to CRAC channelopathy patients, PS exposure and secretion of alpha granules is diminished in mice with platelet-specific deletion of *Stim1* and in chimeric *Orai1^*R*93W/R93W^* animals (Bergmeier et al., 2009; Ahmad et al., 2011). Chimeric *Stim1^–/–^* and *Orai1^–/–^* mice additionally show impaired platelet aggregation and thrombus formation, leading to a slight increase in bleeding time (Varga-Szabo et al., 2008; Braun et al., 2009; Gilio et al., 2010). The murine TAM/STRMK models similarly recapitulate the coagulation defects seen in the patients, as thrombocytopenia is evident in all three STIM1 D84G, I115F, and R304W models (Grosse et al., 2007; Cordero-Sanchez et al., 2019; Silva-Rojas et al., 2019). Further analyses on the STIM1 D84G mice uncovered that the pre-activation state of the platelets increases platelet clearance, and thereby prevents efficient platelet aggregation (Grosse et al., 2007). If and to what extent the bleeding diathesis is exacerbated by the immune system defects in TAM/STRMK mice and potentially in patients remains to be determined.

### Muscle Weakness

Skeletal muscles maintain posture and allow movements under the voluntary control of the somatic nervous system, and also regulate body temperature and nutrition storage. SOCE activation and extracellular Ca^2+^ entry is significantly faster in myofibers compared with other cell types, occurring within milliseconds after each action potential (Launikonis et al., 2009; Edwards et al., 2010). This is believed to be related to the presence of the muscle-specific STIM1L isoform forming pre-activated Ca^2+^ entry units with ORAI1 at the SR/plasma membrane junction (Darbellay et al., 2011). Refilling of the Ca^2+^ stores is mediated by the ATP-dependent SERCA pumps to maintain high Ca^2+^ gradients across the SR membrane, thus limiting the SR depletion of Ca^2+^ during repetitive tetanic stimulations (Pan et al., 2002; Zhao et al., 2005).

CRAC channelopathy patients manifest neonatal hypotonia and generalized muscle weakness, and show delayed motor milestones and reduced walking distance in infancy, with additional respiratory insufficiency in individual cases (Feske et al., 2006; McCarl et al., 2009; Picard et al., 2009; Fuchs et al., 2012; Chou et al., 2015; Schaballie et al., 2015; Badran et al., 2016; Lian et al., 2018). Histological investigations were performed on muscle biopsies from two patients, and revealed fiber type I fiber predominance and type II atrophy (McCarl et al., 2009; Lian et al., 2018). Muscle weakness and exercise intolerance are primary clinical features of TAM/STRMK, and the onset and severity depend on the causative gene and correlate with the position of the mutation (Morin et al., 2020). In most cases, disease onset is during infancy or childhood, and first and foremost affects the proximal muscles of the lower limbs. Muscle weakness is generally accompanied by elevated serum creatine kinase (CK) levels, indicating moderate fiber degeneration, and myalgia and cramps are often observed as secondary features, but can also occur as isolated signs (Bohm et al., 2013; Bohm et al., 2014, 2017, 2018; Hedberg et al., 2014; Misceo et al., 2014; Morin et al., 2014, 2020; Nesin et al., 2014; Endo et al., 2015; Markello et al., 2015; Walter et al., 2015; Barone et al., 2017; Garibaldi et al., 2017; Harris et al., 2017; Noury et al., 2017; Alonso-Jimenez et al., 2018; Borsani et al., 2018; Li et al., 2019; Claeys et al., 2020). Noteworthy, Ca^2+^ overload in skeletal muscle fibers has been shown to disrupt excitation-contraction coupling (ECC) (Lamb et al., 1995), which possibly contributes to the reduced muscle force in TAM/STRMK patients. Muscle sections from affected individuals typically show tubular aggregates appearing in red on Gomori trichrome staining, and adopting a honeycomb structure of densely packed tubules on electron microscopy (Chevessier et al., 2004, 2005; Bohm and Laporte, 2018). The tubular aggregates are highly Ca^2+^-rich, and immunofluorescence studies have shown that they essentially contain SR proteins such as STIM1, calsequestrin, triadin, or RyR1, indicating that they are of reticular origin (Chevessier et al., 2004, 2005; Bohm et al., 2013, 2017; Endo et al., 2015). It has been suggested that the abundance of Ca^2+^ in muscle fibers may cause SR protein misfolding and aggregation, leading to the formation of membrane stacks as precursors of tubular aggregates (Morin et al., 2020). Alternatively, the occurrence of tubular aggregates may reflect the attempt to regenerate a functional triad, a specialized membrane complex in skeletal muscle hosting the ECC machinery. This is supported by the observation that Ca^2+^ excess induces the proteolysis of junctophilins, which tether the SR membrane to deep plasma membrane invaginations known as T-tubules to form the triad (Murphy et al., 2013). Further histopathological signs on TAM/STRMK biopsies encompass fiber size variability, type I fiber predominance, type II fiber atrophy, internalized nuclei, vacuoles, and fibrosis (Bohm et al., 2013, 2014, 2017, 2018; Hedberg et al., 2014; Morin et al., 2014, 2020; Nesin et al., 2014; Endo et al., 2015; Markello et al., 2015; Walter et al., 2015; Harris et al., 2017; Noury et al., 2017; Borsani et al., 2018; Li et al., 2019; Claeys et al., 2020).

*Stim1* KO and *Orai1* KO Mice die perinatally (Baba et al., 2008; Gwack et al., 2008; Oh-Hora et al., 2008), and the muscle-specific invalidation of either gene results in diminished cellular Ca^2+^ transients following stimulation, and interferes with muscle contractility and the production of force (Stiber et al., 2008; Li et al., 2012; Wei-Lapierre et al., 2013; Carrell et al., 2016). The mice also show an increased susceptibility to fatigue (Stiber et al., 2008; Wei-Lapierre et al., 2013; Carrell et al., 2016), and histological and ultrastructural investigations of muscle samples uncovered a reduction in fiber size and overall muscle mass, and swollen mitochondria (Stiber et al., 2008; Li et al., 2012; Wei-Lapierre et al., 2013; Carrell et al., 2016). The STIM1 I115F and R304W TAM/STRMK mouse models exhibit reduced muscle force (Cordero-Sanchez et al., 2019; Silva-Rojas et al., 2019), and continuous muscle stimulation evidenced a slower force decay compared with WT littermates, presumably reflecting an increased resistance to fatigue (Silva-Rojas et al., 2019). The animals exhibit elevated serum CK levels, and histological examinations of muscle samples revealed an increased proportion of type I fibers, an overall reduction of fiber diameter with signs of muscle fiber degeneration and regeneration, and electron microscopy uncovered swollen mitochondria (Cordero-Sanchez et al., 2019; Silva-Rojas et al., 2019). Most strikingly, tubular aggregates are absent from muscles in both murine TAM/STRMK models, highlighting a major structural difference between human and mouse muscle pathologies despite the concordance of the overall clinical picture. Considering the observation that dystrophic signs are more prominent in TAM/STRMK mice than in patients, the tubular aggregates may protect the human muscle fibers from degeneration by bundling excessive free Ca^2+^. Another STIM1 R304W mouse model does not show functional or structural skeletal muscle aberrations (Gamage et al., 2018), and a potential muscle phenotype of the STIM1 D84G mouse was not assessed (Grosse et al., 2007).

## Soce Regulators, Associated Diseases and Animal Models

Ca^2+^ controls a multitude of metabolic processes, signaling pathways, and cellular functions including transcription, proliferation, differentiation, and exocytosis. As a major regulator of Ca^2+^ homeostasis, SOCE takes a central role in the physiology of all tissues and organs, and needs to be adaptable to the Ca^2+^ sensitivity and Ca^2+^ balance of the individual cell types forming an organism.

The STIM1 homologue STIM2 has been shown to modulate SOCE activity (Darbellay et al., 2011; Miederer et al., 2015), and several additional regulators either promoting or restricting extracellular Ca^2+^ entry are known. Positive effectors encompass CRACR2A and septins, facilitating STIM1-ORAI1 coupling (Srikanth et al., 2010; Sharma et al., 2013), STIMATE, favoring STIM1 clustering (Jing et al., 2015), and the inositol triphosphate receptor (IP_3_R), lowering the local Ca^2+^ levels in proximity to the STIM1 EF hands (Sampieri et al., 2018). Negative regulators include SARAF and calsequestrin, both hampering STIM1 oligomerization (Palty et al., 2012; Jha et al., 2013; Wang et al., 2015), Golli-MBP, binding and dispersing the STIM1-ORAI1 complex (Feng et al., 2006; Walsh et al., 2010), ORMLD3, fostering STIM1-ORAI1 uncoupling following Ca^2+^ influx (Carreras-Sureda et al., 2013), and ALG2, ALG14, DPAGT1, GFPT1, and GMPPB, all mediating post-translational modifications repressing the activity of STIM1 and ORAI1 (Belaya et al., 2012, 2015; Guergueltcheva et al., 2012; Cossins et al., 2013). To date, IP_3_R, calsequestrin, ALG2, ALG14, DPAGT1, GFPT1, and GMPPB have been associated with human pathologies, suggesting that mutations in the other SOCE regulators may similarly impact on Ca^2+^ homeostasis and cause CRAC channelopathy, TAM/STRMK, or related disorders. It is, however, possible that potential physiological anomalies remain within the normal range of tissue and organ functionality due to a marginal effect on the intracellular Ca^2+^ balance, and are therefore hardly detectable.

LoF mutations in *ITPR1*, encoding IP_3_R type 1, cause Gillepsie syndrome (GLSP), characterized by muscular hypotonia, mydriasis, ataxia, and intellectual disability (Gerber et al., 2016), and *Itpr1*-null mice manifest severe ataxia and epileptic seizures (Matsumoto et al., 1996). Mutations in *ITPR2* and *ITRP3*, respectively, encoding IP_3_R types 2 and 3, are associated with anhidrosis in patients (Klar et al., 2014; Kerkhofs et al., 2018), and the same phenotype is also observed in *Itpr2* and *Itpr3* double knockout mice (Futatsugi et al., 2005). This is in accordance with the idea that the reduction of SOCE through the loss of the positive effector IP_3_R results in a clinical phenotype resembling CRAC channelopathy. In the same line, *STING* GoF mutations are found in patients with systemic inflammatory syndrome and autoimmunity (Jeremiah et al., 2014), and a mouse model carrying a patient mutation recapitulates the clinical signs (Bouis et al., 2019). STING is a signaling adaptor residing in the ER, and is retained in an inactive state through direct interaction with STIM1. In response to DNA pathogens, STING translocates to the ER-Golgi intermediate compartment to trigger an interferon response through the STING-TBK1-IRF3 pathway (Ishikawa and Barber, 2008, 2011). Loss of STIM1 in mouse and human CRAC channelopathy cell lines induces a spontaneous activation of STING and an enhanced expression of type 1 interferons under sterile conditions, thereby stimulating the immune system even in the absence of pathogens (Srikanth et al., 2019).

Calsequestrin (CASQ1) is the major Ca^2+^ buffering protein in the sarcoplasmic reticulum in skeletal muscle, and polymerizes with increasing luminal Ca^2+^ concentrations (Manno et al., 2017). In turn, Ca^2+^ store depletion promotes depolymerization, and the calsequestrin monomers sequester STIM1 and hence negatively regulate SOCE (Wang et al., 2015). Specific missense mutations in *CASQ1* interfere with the polymerization and depolymerization dynamics of calsequestrin, lower the Ca^2+^ buffer capacities of the reticulum and impair calsequestrin monomerization, leading to an increase in SOCE activity (Barone et al., 2017; Bohm et al., 2018). As calsequestrin expression is restricted to skeletal muscle, patients with *CASQ1* mutations show a mild form of TAM/STRMK with late-onset muscle weakness, myalgia, and abundant tubular aggregates, but without additional multi-systemic signs (Bohm and Laporte, 2018; Bohm et al., 2018). A murine model harboring a *CASQ1* mutation found in patients does not exist, and the total loss of calsequestrin generates a malignant hyperthermia phenotype with an increased risk of sudden death in mice (Protasi et al., 2009). Noteworthy, CASQ1 null mice show an increased expression of STIM1 and ORAI1 associated with enhanced SOCE activity, possibly reflecting a compensatory mechanism to ensure the maintenance of contractile force despite the reduction of bound and releasable Ca^2+^ in the SR (Michelucci et al., 2020). Tubular aggregates containing STIM1 and calsequestrin are also seen on muscle biopsies from patients with limb-girdle congenital myasthenic syndrome (LG-CMS), marked by fluctuating muscle weakness and fatigability (Evangelista et al., 2015). LG-CMS is caused by the impaired transmission at the neuromuscular junction, the relay between motor neuron and muscle fiber, and results from LoF mutations in *ALG2*, *ALG14*, *DPAGT1*, *GFPT1*, or *GMPP8* (Belaya et al., 2012, 2015; Guergueltcheva et al., 2012; Cossins et al., 2013). All five genes code for proteins of the glycosylation pathway and procure posttranslational modifications to a wide variety of proteins including STIM1 and ORAI1. Hypoglycosylation of STIM1 and ORAI1 stimulates SOCE and extracellular Ca^2+^ influx (Selcen et al., 2014), and the muscle-specific deletion of *Gfpt1* in mice causes myasthenia and the occurrence of tubular aggregates in muscle fibers (Issop et al., 2018). These examples show that the dysfunction of proteins directly or indirectly associated with STIM1 and ORAI1 can cause human pathologies overlapping with TAM/STRMK at the clinical and histological level.

## Conclusion and Perspectives

LoF mutations in *STIM1* and *ORAI1* impair SOCE and cause CRAC channelopathy, while GoF mutations in both genes involve SOCE over-activation and result in TAM/STRMK (Lacruz and Feske, 2015; Bohm and Laporte, 2018). In agreement with the opposite mutational effects and pathomechanisms leading to either CRAC channelopathy or TAM/STRMK, both disorders by and large show clinical mirror phenotypes affecting the eyes, bones, immune system, platelets, and skeletal muscle. While CRAC channelopathy is characterized by mydriasis, increased bone mineralization, immunodeficiency, splenomegaly, impaired platelet activation, and muscle hypotonia, TAM/STRMK patients typically present with miosis, decreased bone mineralization, hyposplenism, platelet pre-activation, and muscle cramping. A single TAM/STRMK patient was additionally diagnosed with lymphoproliferation (Sura et al., 2020), indicating an over-active immune system. Investigations on TAM/STRMK mouse models confirmed a dysregulation of various immune system cells, which may account for the skin phenotype in humans and mice (Silva-Rojas et al., 2019). It is interesting to note that the clinical anomalies of platelets and skeletal muscle are similar in CRAC channelopathy and TAM/STRMK patients despite the inverse pathogenic effect of *STIM1* and *ORAI1* LoF and GoF mutations at the molecular level, highlighting the importance of strict SOCE regulation for normal tissue physiology. Thrombus formation is impaired in both disorders and enhances the tendency to bleed following injury. This is due to the reduced activation of platelets in CRAC channelopathy (Nakamura et al., 2013), and results from the impaired adhesion between platelets in TAM/STRMK (Markello et al., 2015). Similarly, muscle weakness either arises from the incapacity to sustain a sufficient muscle tonus in the absence of Ca^2+^ store refill in CRAC channelopathy, or from cytosolic Ca^2+^ excess disrupting excitation-contraction coupling and/or restraining proper muscle relaxation in TAM/STRMK.

CRAC channelopathy and TAM/STRMK mouse models recapitulate the main clinical signs of the human disorders, and are valuable and powerful tools to understand the importance of Ca^2+^ balance and the impact of Ca^2+^ imbalance on eye, bones, enamel, skin, platelets, spleen, immune system, and skeletal muscle physiology. Patients are usually examined by specialized physicians with a major focus on the principal handicap, and additional phenotypic anomalies might be overlooked, especially in the context of multi-systemic disorders with mild to moderate involvement of specific tissues. In contrast, murine models generally undergo unbiased phenotyping and offer the possibility for a detailed analysis of all organs to provide an overview of the disease. As an example, the complete characterization of the STIM1 R304W TAM/STRMK mouse model unveiled anomalies of the glucose metabolism, hepatic function, and the immune system (Silva-Rojas et al., 2019), which have not been described in patients yet, but might be of medical importance. Conversely, psychiatric diseases including confusion (Misceo et al., 2014; Harris et al., 2017), Capgras syndrome (Harris et al., 2017), and manic psychosis (Harris et al., 2017) have only been reported in individual TAM/STRMK cases, and thorough investigations on the mouse model might help to determine if these anomalies are disease-related or unrelated. Lastly, the mouse models faithfully recapitulating CRAC channelopathy and TAM/STRMK can serve for the assessment of therapeutic approaches, which may also be relevant for other Ca^2+^-related disorders affecting the bones, platelets, spleen, immune system, or skeletal muscle.

## Author Contributions

RS-R and JB wrote the manuscript. All authors contributed to the article and approved the submitted version.

## Conflict of Interest

The authors declare that the research was conducted in the absence of any commercial or financial relationships that could be construed as a potential conflict of interest.
